# Supplementary figures and images for: De novo spatiotemporal modelling of cell-type signatures in the developmental human heart using graph convolutional neural networks
Source: PLoS Comput Biol. 2022 Aug 12;18(8):e1010366. doi: 10.1371/journal.pcbi.1010366 (PMC9401155; doi:10.1371/journal.pcbi.1010366)

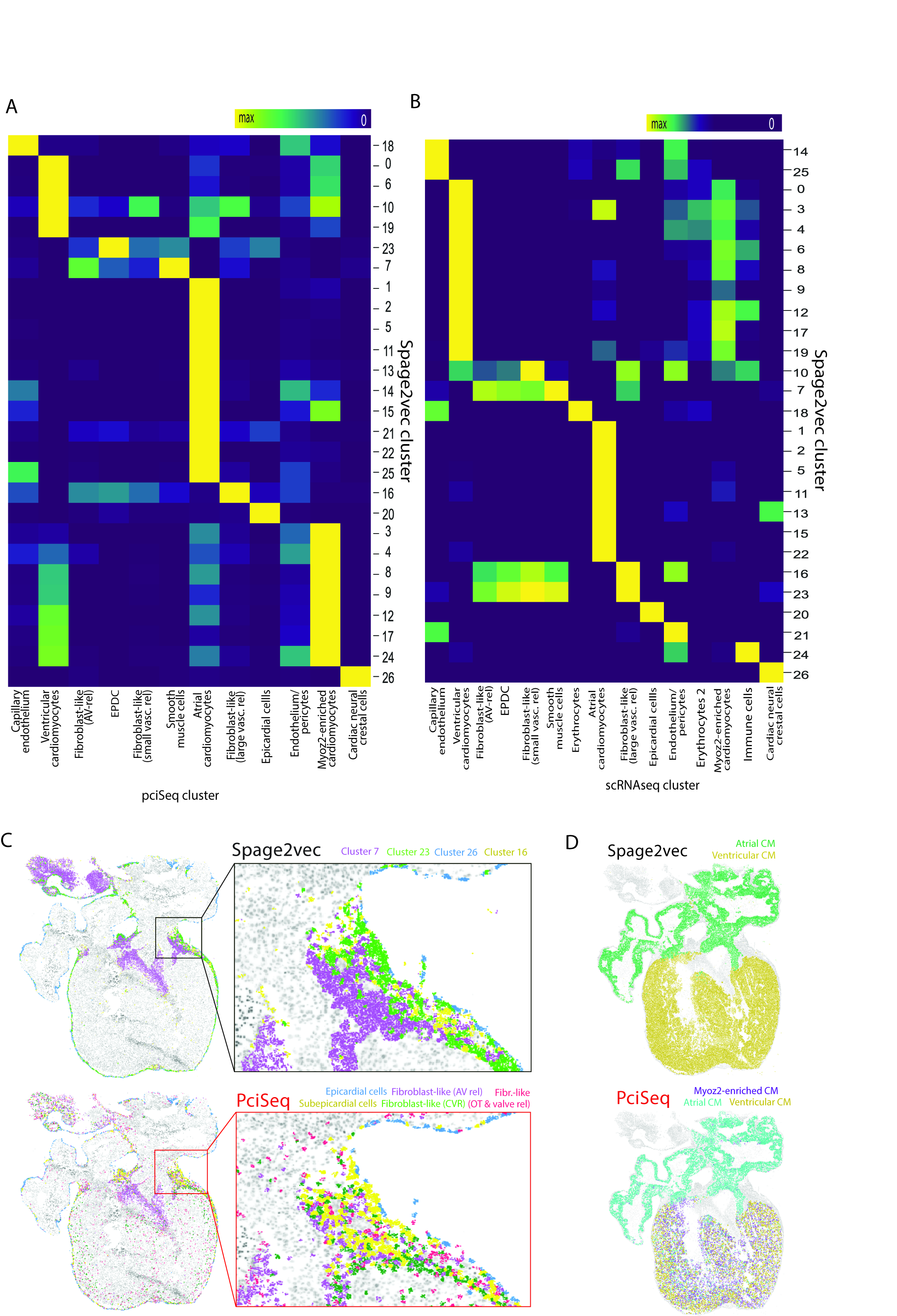

Supplement: S1 Fig — A. Heatmap representing the confusion matrix between the cell type assigned to each read via pciSeq in Asp et al. [7] and the spage2vec cluster annotations. B. Heatmap representing the correlation between the expression profile of each spage2vec cluster and each cell type described using scRNA-seq in Asp et al. [7] for the 69 genes included in both datasets. C. Spatial location of a subset of clusters from the spage2vec analysis (top) and pciSeq (bottom) in a specific sample from pcw 6.5. Clusters selected represent both epicardial cells and fibroblast-like cells /epicardium derived cells in both cases and colors have been based on the similarities between spage2vec clusters and pciSeq clusters. A zoomed in region is shown for both datasets. D. Spatial location of cardiomyocyte-related clusters defined by both spage2vec (top) and pciseq (bottom) in a specific section from pcw 6.5. Each spage2vec cluster assigned to cardiomyocytes were classified as atrial or ventricular according to their molecular signature in Fig 1C. (TIF) [file pcbi.1010366.s001.tif]

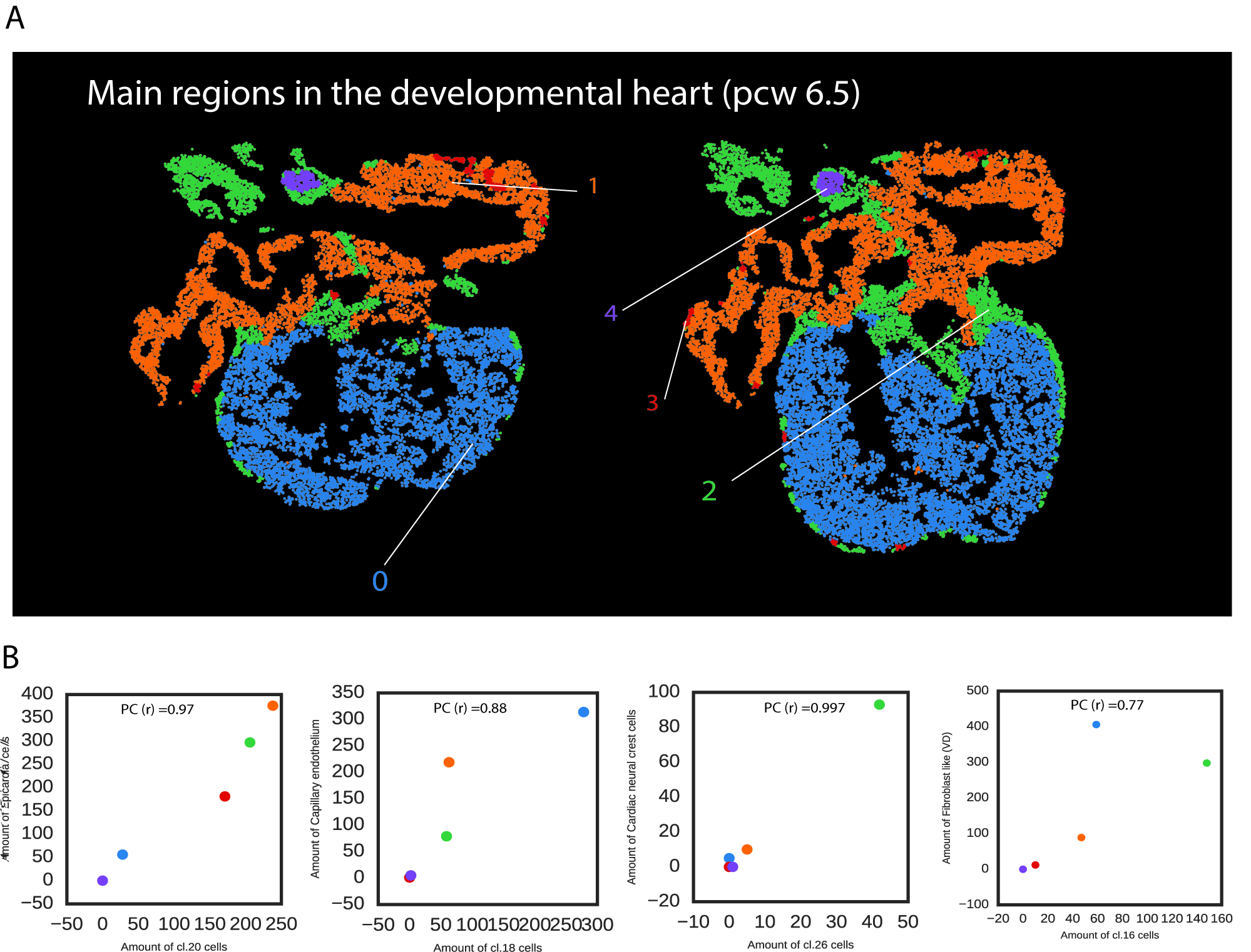

Supplement: S2 Fig — A. Map of the main morphological regions identified in pcw6.5 sections. Regions were calculated by redefining every read based on the reads present in a radius of 70 pixels/22.8 um to capture the main tissue domains and applying leiden clustering on it. B. Scatter plot representing the abundancies of specific paired spage2vec-pciseq clusters in the regions defined in S2A (from left to right: epicardial cells-cluster 20; capillary endotheium-cluster 18; cardiac neural crest cells-cluster 26 and Fibroblast-like (VD)-cluster 16). Pearson correlation for every pair of clusters is included in the scatter plot. (TIF) [file pcbi.1010366.s002.tif]

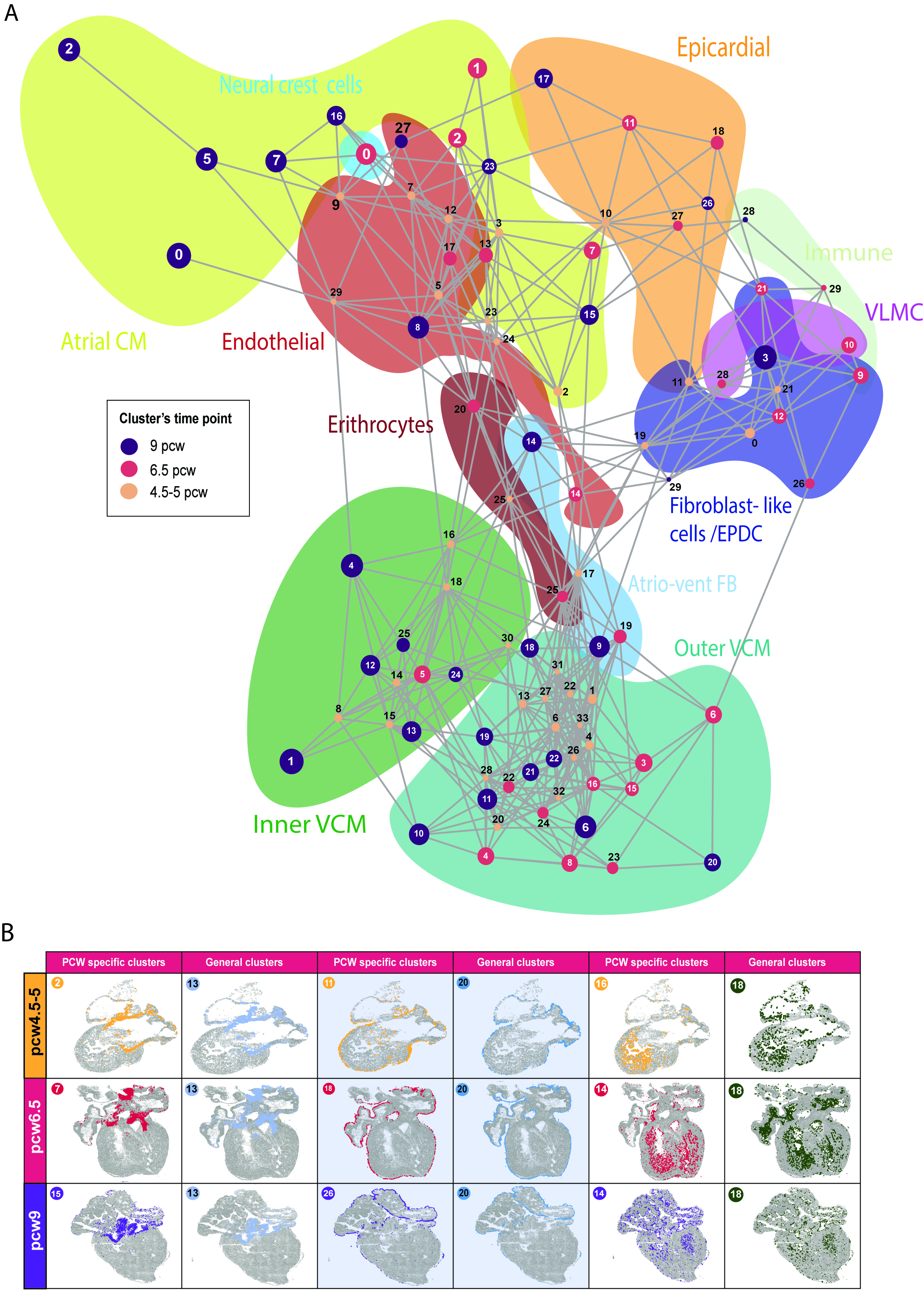

Supplement: S3 Fig — A. PAGA plot representing all clusters found in the time-point specific analyses of pcw 4.5–5, pcw 6.5 and pcw 9. Each cluster is represented in a node and backg//round colors indicate main cell type annotations. B. Spatial location’s comparison between general clusters (Figs 1, S1, S2 and S3) and time-point specific clusters. Three main clusters are represented: cluster 13 (left), cluster 20 (middle) and cluster 18 (right) in one of the samples of each time point, together with their correspondent time point-specific cluster. (TIF) [file pcbi.1010366.s003.tif]

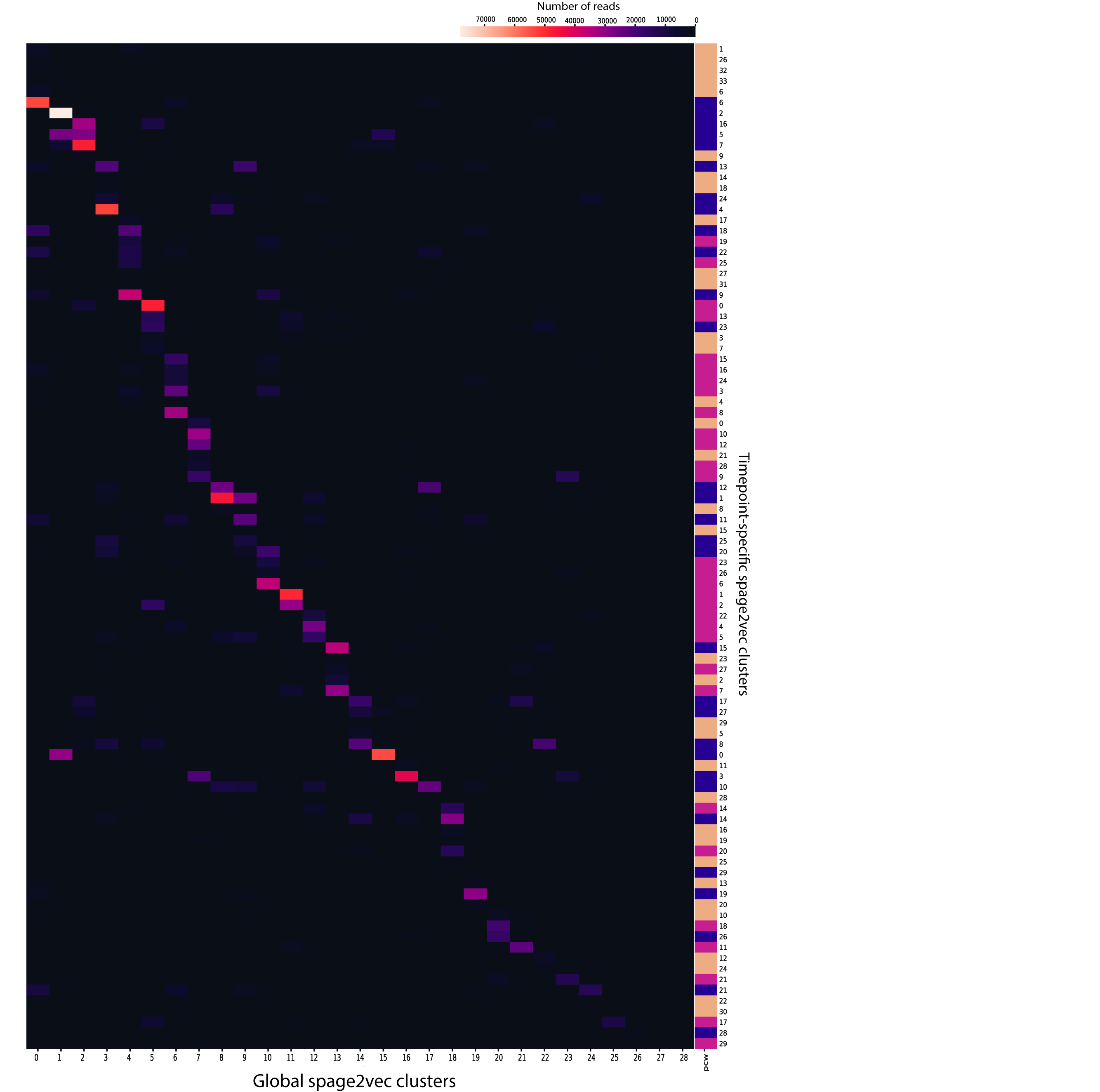

Supplement: S4 Fig — Heatmap representing the confusion matrix between the cluster assigned to each read in the general analysis (Figs 1, S1, S2 and S3) and the cluster assigned to each read in the time point-specific analysis. Color column situated next to the time-point specific cluster labels indicates the time point where each cluster has been detected. The color code used in Fig 1A is used to label each time point. (TIF) [file pcbi.1010366.s004.tif]

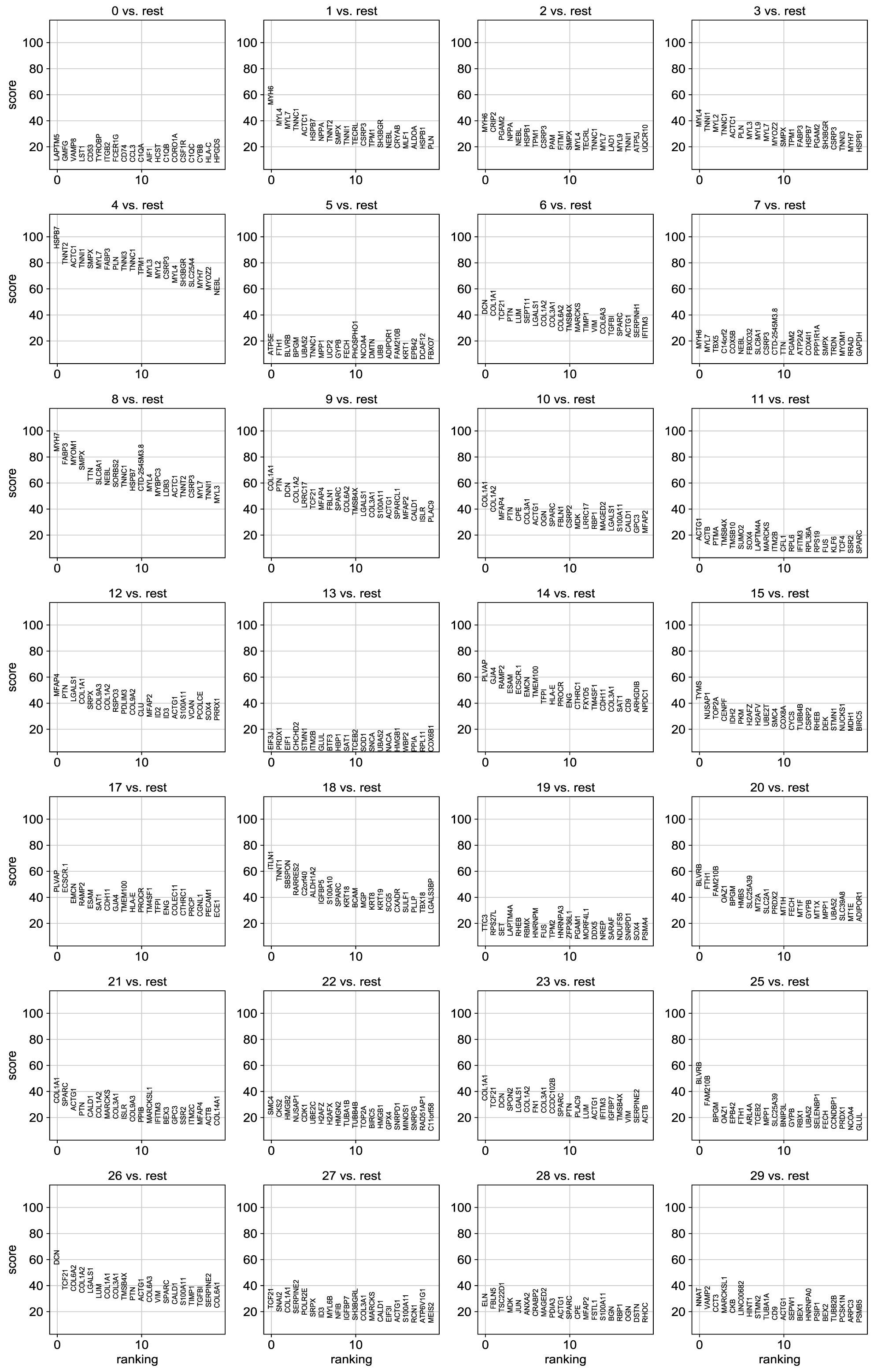

Supplement: S5 Fig — Top 15 differentially expressed genes for the clusters found in the individual analysis of pcw 6.5. Scores of each gene (y axis) corresponds to the Wilcoxon rank-sum test score. (TIF) [file pcbi.1010366.s005.tif]

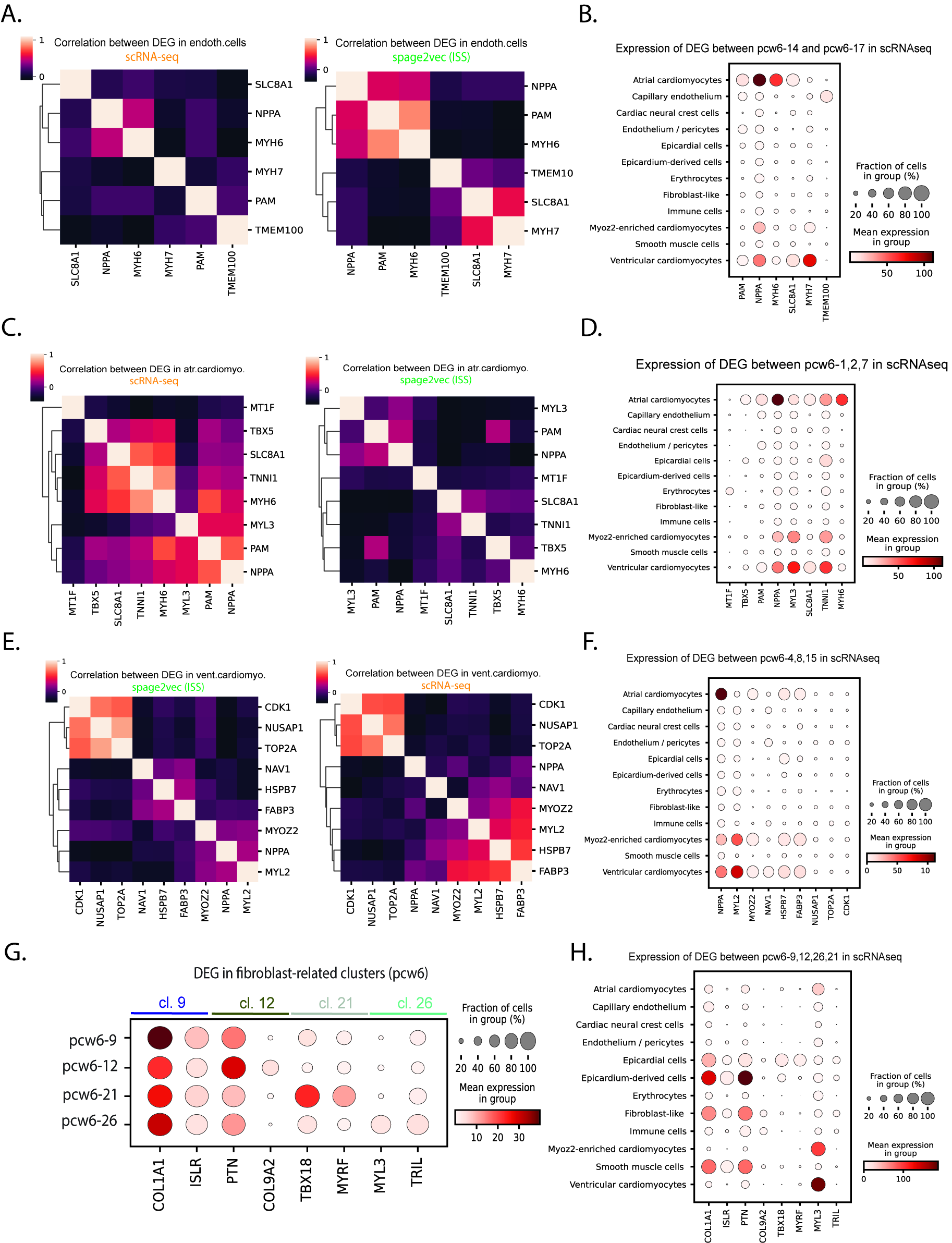

Supplement: S6 Fig — A. Heatmap representing the correlation patterns found in scRNAseq (left) and spage2vec (right) between the three most differentially expressed genes (DEG) between endothelium-related clusters represented in Fig 3A. B. Dot plot representing the expression detected via scRNAseq of the three most DEG between endothelium-related clusters in the cell types identified in Asp et al. C. Heatmap representing the correlation patterns found in scRNAseq (left) and spage2vec (right) between the three most DEG between atrium cardiomyocytes subclusters represented in Fig 3A. D. Dot plot representing the expression detected via scRNAseq of the three most DEG between atrium cardiomyocytes subclusters in the cell types identified in Asp et al. E. Heatmap representing the correlation patterns found in scRNAseq (left) and spage2vec (right) between the three most DEG between ventricular cardiomyocytes subclusters represented in Fig 3A. F. Dot plot representing the expression detected via scRNAseq of the three most DEG between ventricular cardiomyocytes subclusters in the cell types identified in Asp et al. G. Dot plot representing the expression of the 2 most DEG of each fibroblast/EPDC subcluster (cl) H. Dot plot representing the expression detected via scRNAseq of the top DEG between fibroblast/EPDC subclusters in the cell types identified in Asp et al. (TIF) [file pcbi.1010366.s006.tif]

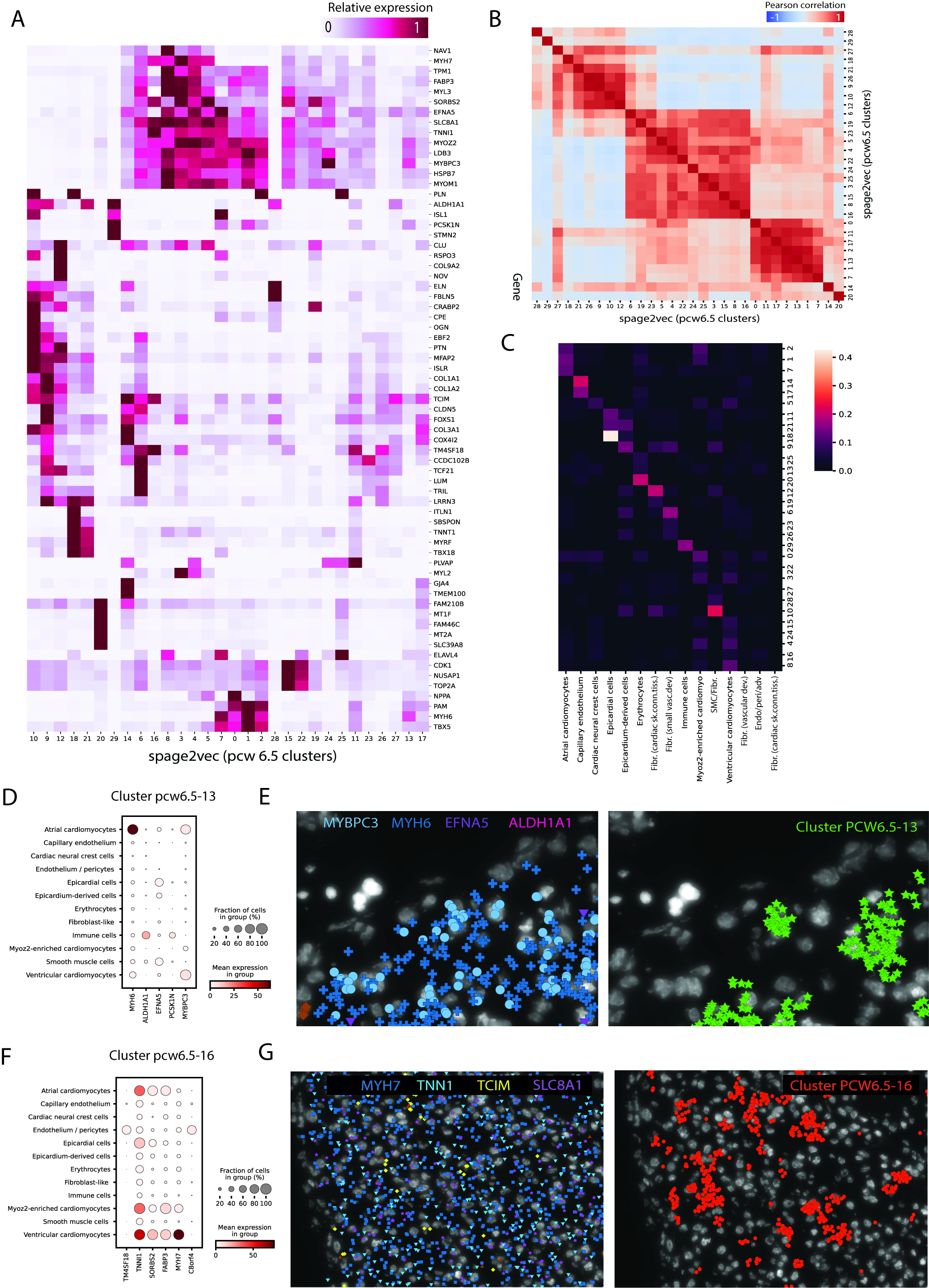

Supplement: S7 Fig — A. Heat map representin the mean expression of each spage2vec pcw6.5 cluster for the genes included in the ISS panel. B. Heat map representing the cross correlation of the spage2vec pcw6.5 clusters based on their mean expression. C. Jaccard index derived from the integration of the scRNA-seq dataset and the spage2vec pcw6.5 clusters D. Dot plot representing the expression of the genes expressed in the spage2vec pcw6.5–13 cluster in the different scRNA-seq cell types. E. Region of interest of the in situ sequencing samples (pcw6.5) representing the location of cluster pcw6.5–13 (right) and some of genes expressed in this cluster (left) F. Dot plot representing the expression of the genes expressed in the spage2vec pcw6.5–16 cluster in the different scRNA-seq cell types. G. Region of interest of the in situ sequencing samples (pcw 6.5) representing the location of cluster pcw6.5–16 (right) and some of genes expressed in this cluster (left) (TIF) [file pcbi.1010366.s007.tif]
