# Supplementary material for: De novo spatiotemporal modelling of cell-type signatures in the developmental human heart using graph convolutional neural networks
Source: PLoS Comput Biol. 2022 Aug 12;18(8):e1010366. doi: 10.1371/journal.pcbi.1010366 (PMC9401155; doi:10.1371/journal.pcbi.1010366)
Supplement: Attachment — Submitted filename: Spage2vec_reviewer_PLOS_commbio_response.docx [file pcbi.1010366.s008.docx]

**Response to the reviewer’s comments**

*Dear editors,*

*We are pleased to see the positive feedback from the reviewers and the suggested revision in order to improve the manuscript. We have now prepared a revised version of the manuscript titled “De novo spatiotemporal modelling of cell-type signatures in the developmental human heart using graph convolutional neural networks” that takes all suggestions into account.*

*According to reviewers’ suggestion, we have revisited some of the aspects of the manuscript and clarified some of the results. This include the reannotation of some of the clusters in Figure 1 and 2; the exploration of the biological relevance of some of the clusters presented in our analysis, (main text, S2, S6 and S7); and a deeper description of the integration of scRNAseq and ISS data done using SpaGE (S7C). We have made all the data and code available for further exploration, as suggested by Reviewer 2. In addition, we have corrected the grammatical inconsistencies present across the manuscript and we have modified the main and supplementary figures according to Reviewers’ suggestions. Finally, we have addressed all the concerns risen by both reviewers and given an answer to their questions, as you can find in this letter. Note that the reviewer’s points are written in black, while the answer give is included in blue. We have included, as required, two versions of the manuscript: one with the modifications highlighted in red, and a second version where modifications are not highlighted.*

**Reviewer #1:**

Graph neural networks has recently become a very widely used and powerful method for analyzing diverse complex networks, e.g., social network, financial network, protein networks, brain networks [1]. The paper applied the Spage2vec method, an advanced graph neural network model, to effectively characterize the cell types at sub-cellular imaging resolution for the development of fetal heart from 4.5 to 9 post conception weeks (PCWs)---three developmental stages. However, the in situ pciSeq technique published in 2020 only explored cell diversity for the 6.5 PCW heart development stage, yet not considered the 4.5-5 PCW and 9 PCW time points.

Spage2vec requires no cell segmentation, and can learns the latent cell expression representations by incorporating the critical spatial molecular organization structure property. Using the obtained latent cell representations, it enables to characterize previously unreported molecular diversity within cardiomyocytes and epicardial cells. Moreover, it can identify their characteristic expression signatures for diverse downstream tasks, e.g., subpopulation detection. The experimental results show that the proposed method enables to discover 27 meaningful cellular clusters (more than the 12 cell types defined in the scRNA-seq data) shared across the three different heart development embryonic stages.

The authors also built an open-source GitHub code repo for their work and provided an online platform for interactively visualization of multiple cell type clusters in different colors. Having public repo and online visualization platform can greatly aid other researchers in this field in better understanding the method implementations and the entire study.

After reading through the paper, I listed some of my comments and questions below.

Page 3, Line 70-71:

If I understood well, the study on the pciSep technique was initially developed for brain cortex, yet not for the development of the heart. However, the author described that “the overall study was the first comprehensive spatial atlas of the developmental human heart”.

The spatial atlas of the developmental human heart was published by Asp et al. and despite not introducing pciSeq as a method it provided the first comprehensive spatial cell atlas of the human heart development to our understanding. In the original pciSeq article (Qian et al. 2020) pciSeq is described as a method to assign scRNAseq defined cell types to specific cells in situ, and the mapping of interneurons in the CA region of mouse hippocampus was clearly presented as an example application where there is a ground truth with respect to the expected location of the different subtypes of interneurons. However, it’s not organ-specific and can be applied to any organ from which there is available scRNAseq data.

The Asp paper provides, on the other hand, was the first comprehensive spatial cell atlas of the human heart development (to our understanding). The Asp study is however limited: probabilistic cell typing by pciSeq was only possible for the 6.5 pcw developmental stage where single cell data was available, leaving the cellular diversity in the 4.5-5 and 9 pcw time points unexplored at a single cell level. This is where the current manuscript contributes by enabling cell typing also at the 4.5-5 and 9 pcw time points using spage2vec.

Page 7 Line 147:

There is a typo in the sentence “clusters whith (typo) distinct and consistent spatial…”.

This typo has been corrected in the updated version of the manuscript.

Material and Methods:

• Page 13, Line 301/304: Does Spage2vec can capture global graph structure property, i.e., second-order proximity, or just capture local neighborhood relations among mRNA spots?

Spage2vec can capture different neighborhoods depending on the distance used to define the graph. Therefore, it can be adjusted to capture subcellular, cellular or supracellular patterns. This is explained in lines from 245 to 259. Briefly, each node is connected by edges with a maximum distance of = 44.9 pixels, corresponding to 14.58 μm. This was set automatically by finding a maximum distance such that 99% of all nodes in the graph are connected to at least one neighbor. Moreover, the spage2vec graph neural network have two graph convolution layers through which second-order proximity is considered by propagation information for each node from its neighbors and the neighbors of the it neighbors.

• Line 311, page 14: I would suggest to add more details about the number of nodes in the constructed graph, and what is the node feature dimension? What is the adjacency matrix dimension in the constructed graph? weighted or unweighted, directed, or undirected?

The number of nodes is 99% of the number of reads; all reads apart from very isolated reads are included (maximum distance is set such that 99% of all nodes in the graph are connected to at least one neighbor). The feature dimension is the number of different targeted genes in the dataset. The graph is undirected and not weighted. Adjacency matrix number of nodes squared, but it is very sparse.

• What are the performance if directly apply conventional clustering t-sNE or IsoMap

The purpose of the graph neural network is to create a descriptor of local neighborhoods, that can be presented as a manifold in a multi-dimensional space, where similar local neighborhoods are close in this multi-dimensional space, but not necessarily close in physical space. E.g., all mRNAs belonging to the same cell-type will be close in the manifold, but not necessarily be physically close in the heart tissue. We chose to apply UMAP to the manifold created by the graph neural network since it is a stable and reproducible clustering method. A similar performance would likely have been achieved depending on the initialization of t-SNE (which has proven by others to perform similarly to UMAP, see Becht, E. et al.(1) .Since the raw spatial data does not group similar cell types, a direct application of t-SNE or IsoMap (or UMAP) would lead to a completely different type of clustering.

• Is there any correlation between the three different development stages? If has, is it possible to learn these evolving dynamics information directly using spage2vec model? or using Spage2vec to capture both spatial and temporal graph embeddings in the latent space simultaneously?

The manifold created by spage2vec shows clear clusters as well as gradients, suggesting slow transitions in gene expression when one cell type develops into a different cell type. One could potentially explore these changes by combining them with a temporal axis, but ideally one should collect data from more than the three timepoints presented here.

**Reviewer #2:**

Dear authors,

I have reviewed the manuscript titled “De novo spatiotemporal modelling of cell-type signatures in the developmental human” presented by Salas et al. In their work they present their re-analysis of the human developmental heart spatial data presented by Asp et al 2019 using a graphical modelling approach that is independent of identifying cells. In doing so, they identify previously undescribed molecular diversity within cardiomyocytes and epicardial cell populations and describe gene expression trajectory dynamics within the tissues.

Both the methodolocial approach and results are indeed very interesting, and I would like to see them published. Cell segmentation free approaches are still underappreciated in spatially resolved transcriptomics data analysis. However, I feel that the paper is currently lacking investigation and validation of the differences between the obtained results, and those presented by Asp et al. I hope that I can elaborate on my concerns with the following points.

Major

1) Comparative analysis of the spage2vec and pciSeq/scRNAseq data seems lacking in places. Some specific points regarding this issue are:

1a) Methodological differences leading to different cluster numbers. In scRNAseq it is well established that the clustering parameters play a major role in how many clusters are identified. How invariant are the 27 clusters to parameters used by spage2vec (line 120)? How invariant are the 12 clusters to parameters used by pciSeq? How invariant are the 15 clusters to parameters used by scRNAseq? The authors should investigate that reasonable parameter changes in e.g. the scRNAseq cluster do not result in e.g. 27 clusters that match the spage2vec clusters perfectly (.. which is unlikely, but it should be explored). If the aut

In order to address this point, we would first like to clarify that discrepancies in the number of clusters identified by scRNA-seq and spatially by pciSeq are due to the lack of identification of cells with a high probability of being (1) Immune cells, (2) Erythrocytes and (3) Erythrocytes 2 in the analysis performed by pciSeq. This means that, although pciSeq tries to classify the cells among the 15 cell types identified by scRNA-seq, only 12 of these 15 signatures were assigned to at least one cell. As a consequence, in the rest of the response we will consider the pciSeq and the scRNA-seq cell types as one unit, since the spatial clusters rely on the definitions found by scRNAseq. We have modified the text accordingly to clarify this point.

The granularity of the clustering both in single cell RNAseq approaches and in spatial ones such as the ones described in this work is known to affect the final outcome and interpretation of the results. In this sense, a deeper resolution in both the scRNAseq datasets and spatial datasets can result in a higher number of potentially biologically meaningful clusters. However, increasing the granularity could also result in overclustering. This is, in essence, forcing a single biologically meaningful cluster to split into two clusters based on minor differences in expression. To address this, we first explored the scRNA-seq dataset obtained from pcw 6.5 and increased the clustering resolution, resulting in a total of 24 clusters. This is presented in Additional Figure 1, an appended Additional Figure that the reviewers can find at the endo this document. Similar to spage2vec’s clusters, the new clusters identified corresponded to subpopulations of endothelial cells, fibroblasts and cardiomyocytes. Indeed, this cardiomyocyte diversity has been recently reported by Sylven et al. 2022(2) in a recent preprint.

Together with this, one of the reasons for identifying more clusters in spage2vec could be the use of two additional time points in the analysis apart from pcw6, which could include new cell types. We identified 4 clusters (1,2,15,27) mostly associated with atrial cardiomyocytes and 2 clusters (3,8) associated with ventricular cardiomyocytes that didn’t present a significant proportion of cells in pcw 6.5. These clusters presented a high abundance at pcw 9 and were characterized by a higher expression of NPPA, which seems to increase its expression in cardiomyocytes through development.

Finally, we’d like to highlight that scRNAseq methods could include a bias in the cell types and proportions estimated from a tissue. This could be caused by problems in the dissociation step or by its actual sequencing coverage, which, at least in the case of 10X scRNA-seq, could not be deep enough to grasp subte changes in cell state. In this sense, spatial methods should provide a more accurate representation of the frequencies of the different cell types present in a tissue.

All in all, we could explain the additional number of clusters as a combination of (1) increased granularity, (2) additional developmental stages used and (3) potential discrepancies between scRNAseq and spatial methods.

1b) I would expect that for the 6.5 PCW dataset (where matched scRNAseq and ISS data exist) that the cell types obtained between the spage2vec can be found in the scRNAseq data. The authors postulate that the detection of additional clusters in the ISS data using spage2vec is due to sampling more cells - this would explain the difference for rare cell types, but would also implicate that cell types with many cell should be equally represented in both the scRNAseq and ISS data. However given that such a large proportion of those cells are seen in the spage2vec data and not in the spage scRNAseq analysis makes it unlikely that this arises for differences in cell number. For example, the spage analysis in Figure 2 shows that a number of spage2vec e.g. clusters 16, 24 ,19 , 23 , 13, 25 and 3 do not appear to have matches in the spage analysis of the scRNAseq data (fig 2B). However, nearly all of these spage2vec clusters have have many cells annotated in the tissue (fig s2) at 6.5PWC. In light of this, how do the authors explain this discrepency?

We would like to remark that spage2vec doesn’t segment individual cells, so a quantification of the cell type frequencies based on the reads assigned to each cluster would assume that all cells contain the same number of reads, which, especially in a targeted approach, is not a valid assumption. Therefore, despite the comparison of reads assigned to each cluster can be a good qualitative measure of the frequency of the clusters, we have avoided quantifying the cell content in the tissues in this way and instead focus the attention on cell location.

We agree, as exposed by Reviewer 2, that a clarification regarding the absence of scRNA-seq cells matching the pcw-6 clusters 16, 24, 19, 23,13, 25 and 3 is needed for the better comprehension of the manuscript. Figure 2 presents the integration of the cells characterized by scRNA-seq and the clusters identified by spage2vec using the ISS datasets as an input. Every cell in the scRNAseq dataset is linked to the n most similar cells from the spatial dataset (in this case spage2vec reads) and its most similar spage2vec cluster is calculated by calculating the frequency of each spage2vec cluster among the top n most similar spatial cells ( in this case reads). This means that we link scRNAseq cells to spage2vec clusters. This justifies that some spage2vec clusters just match few scRNA-seq cells. In the case of those clusters, due to small differences in expression, their most similar scRNAseq cell type matches completely another spage2vec cluster. As a consequence we can’t interpret unmapped spage2vec clusters as new clusters not detected in scRNA-seq. Instead, we can describe those clusters as subclusters of a larger cell population that match worse the cells isolated by scRNA-seq.

To probe this point, we used again Spage to assign every spage2vec read/pseudo-cell to its closer scRNA-seq cell type and we present the analysis in S7 Figure C. Using this alternative approach we observe that indeed clusters pcw6.5-25 corresponds to erythrocytes, pcw6.5-23 And pcw6.5-19 map mostly to fibroblast-like cells while pcw6.5-3 and pcw6.5-24 has a strong association with ventricular and Myoz2 cardiomyocytes. Finally, pcw6.5-16 and pcw6.5-13 do not present a clear similarity to any of the cell types described using scRNA-seq, mapping mostly to cardiomyocytes and erythrocytes respectively, but presenting considerable scores for some additional cell types. In the context of Spage, where a total correspondence between scRNA-seq and the spatial method integrated is expected in terms of cell frequency and cell types identified, the two spage2vec clusters that do not match match well any cell type could represent (1) a new cell type not captured via scRNA-seq or (2) a mixture of cell types.

In order to find this, we identified the top expressed genes in each of both clusters and check its expression in the scRNAseq. We also explored how the expression of these genes was in the tissue, especially in the cells assigned to these clusters. This work is now summarized in S7 Figure D-G. We observed that cluster pcw6.5-13 expressed genes present in a wide range of clusters in scRNAseq. Furthermore, we didn’t identify an enriched coexpression of any of those genes in the reads assigned to pcw6.5-13 in situ, suggesting that this cluster might not represent a true cell type, but rather a mixture of cell types that are located close to each other in the space. In the case of pcw6.5-16, cardiomyocytes and endothelial-related genes were the most expressed genes in the cluster. To check if we were able to identify cells with mixed phenotype, we explored the expression of these genes in situ in the reads assigned to cluster pcw6.5-16. Unfortunately, very distinct patterns for endothelial cells and cardiomyocytes were present in those regions, leading us to the conclusion that this cluster might be a consequence of overclustering cardiomyocytes and taking into account the expression of neighboring endothelial cells in the process. The text has been modified to summarize these findings.

1c) If the authors cannot recapitulate the novel spage2vec cell types in the scRNAseq data, the authors need to explain why this is the case, with the support from other studies or by validation.

Taking pcw6.5 as the only time point that can be compared to a reference scRNA-seq dataset, our study identified diversity within several cell types: endothelial cells, cardiomyocytes, fibroblasts and epicardial cells. We have now included comparisons between the gene expression heterogeneity found within these cell types in S5 Figure. This analysis enabled us to distinguish true molecular diversity, for example, the one identified within atrial and ventricular cardiomyocytes, from clusters that presented different expression levels in our analysis that could not be recapitulated in scRNAseq. Within this category, endothelial cells is the most remarkable case, where the differences in expression between the two endothelium-related clusters (pcw6.5-14 and pcw6.5-17), located in the atria and in the ventricles respectively, matched with differences in expression between atrial and ventricular cardiomyocytes, indicating that both clusters belong to the same cell type in a different spatial context. Despite this type of heterogeneity doesn’t describe the true molecular diversity within a cell type, we consider this classification to be useful to identify similar cells in different spatial contexts that could, eventually, differentiate in two different subpopulations due to differences in their spatial context.

In the case of fibroblasts and epicardial cells, we identified 4 subclusters (pcw6.5-12,21,26 and 9). The top differentially expressed genes within those clusters were mostly expressed in fibroblast/epicardial cells in the available scRNAseq indicating that the effect of neighboring cell types in the definition of these subclusters is limited (S5 Figure G-H), with the only exception being MYL3, differentially expressed in pcw6.5-26, which presents a higher expression in cardiomyocytes. However, even in this case, the other DEG genes in this cluster (i.e. TRIL) present a clear expression pattern matching with the location of cluster pcw6.5-26 *in situ,* indicating that it represents the molecular diversity within fibroblasts.

1d) I find the consistency of the analysis of the Spage scRNAseq clusters (e.g. presented in figure 3A, S4E, S7) to be confusing, as the scRNAseq cluster variability do not correspond to spage2vec clusters variability. An example of how this can lead to confusion is that in cluster 14 and 17 have very different gene expression (e.g TCIM in cluster 14, and MYL2 for 17) in figure 1C, however, neither of these genes appear in the most variable genes for 14 and 17 in Figure 3A, but instead the genes shown in 3A seem to not be very variable at all. How do the authors explain this?

Since there is only single cell RNA sequencing data available for pcw6, the analysis and results presented in Figure 2 and Figure 3 correspond to the sub clustering of only the reads detected in pcw6 (presented in Figure 2A, middle). Therefore, the comparison between clusters pcw6.5-14 and pcw6.5-17 is done because both are associated with endothelial cells in Figure 2B. In addition to that, differentially expressed genes were detected for each of the clusters identified in the analysis of only pcw6 sections in Figure 3A. As Reviewer 2 mentioned, the genes presented in Figure 3A show similar expression patterns for different clusters classified as potential subtypes in the manuscript, as in the case of clusters pcw6.5-14 and pcw6.5-17. This is because, in this case, differentially expressed genes are calculated by comparing the expression of each cluster against all the other cells. Therefore, as it’s shown in this case, the most differentially expressed genes will be general endothelial markers for both clusters and, as a consequence, differences between subclusters are not highlighted. To overcome this limitation, we have now explored differentially expressed genes by only comparing the expression clusters classified within the same cell type. This is, when applied to this example, identifying differentially expressed genes between clusters pcw6.5-14 and pcw6.5-17. This type of analysis highlights differences between clusters with a similar expression and can help to understand better the new clusters detected in our analysis. We also performed this analysis to further explore the diversity within atrial and ventricular cardiomyocytes and we have summarized the results now in S5 Figure. The outcome of this analysis has been clarified in the text and further explored in the answer given to point 1b)

1e) While spage2vec resolves much more sub-cell-types/state in e.g. atrial cardiomyocytes, it does not capture “Fibroblast-like (AV-rel)”, “Endothelium/ pericytes” (Figure S4A), “Erythrocytes 2”, or “Myoz2-enriched cardiomyocytes” very well. This is partially contradictory to the statement of resolving cell type heterogeneity better than scRNAseq (lines 145-147). The authors should clarify the implications of this on this current study, and critically assess the previous cell-typing in Asp et al. Looking at figure S4D, it seems that Spage2Vec misses the visual enrichment of “Myoz2-enriched cardiomyocytes” in the inner part of the left ventricle (and to lesser extent in the right ventricle). The authors should explain how the spage2vec clusters are an improvement over the pciseq clusters in figure S4D.

A key point in any targeted spatially resolved transcriptomics method is the panel selection. An inaccurate panel selection could make two different cell types unresolvable. In order to test if that’s the case in this situation, we took the original scRNA-seq dataset, clustered it to identify the main populations present on it and re-clustered it just considering the markers used in the ISS datasets explored. The logic behind this is that, if a clearly identifiable cluster is no longer identifiable when using solely the gene panel used in ISS experiments, it will not be possible to identify it in the ISS experiment due to the design of the experiment.

Unfortunately, the available full scRNA-seq dataset from Asp. et al doesn’t present the annotations used in the publication, grouping the both Erythrocyte-related clusters in a single cell type and all fibroblast-like subtypes into Fibroblast-like cells. However, when performing leiden clustering on the original scRNA-seq we could detect two clear populations associated with Erythrocytes and some subpopulations within Fibroblast-like cells. When using only the genes present in the ISS panel to cluster the scRNA-seq dataset, we observed that the clusters of Erythrocyte cells weren’t clear any longer, being confused, in this case, with other cells like Neural crest cells (Additional Figure 1,see below). In addition, Erythrocytes 2 is one of the cell types that wasn’t identified via pciSeq, suggesting that, in fact, we might not have cells in the sections analyzed with that particular signature.

The same occurred with Myoz2-enriched cells, which presented a much more similar pattern to ventricular cardiomyocytes when using only the ISS genes. In this sense, the lack of identification of these distinct two populations could be caused by the panel used to characterize them in situ. Furthermore, a recent preprint by Sylven et al.(2) characterized Myoz2-enriched cardiomyocytes cells as part of the ventricular cardiomyocytes’ population. Specifically as a subpopulation of the clusters described as High G2M and Exosome-enriched cardiomyocytes. In that context, the original pciseq results presented in Asp et al.(3) place some Myoz2 enriched cells in the atrium, in disagreement with both the results presented in this manuscript and in Sylven et al., where Myoz2-positive cells are part of ventricular cardiomyocytes.

1f) How was the spatial concordance of cell types determined (line 127-128)? This should be performed statistically.

In order to quantify the spatial concordance of the cell types mentioned in the text, we segmented the datasets into morphologically-relevant regions. Since the morphology of the heart can be quite complex, we benefit from the spatial profiles and redefined every read according to its neighbors but, in this case, considering the global environment (radius=70 pixels/22.8um). By performing superficial clustering, we identified the main regions in the heart sections characterized, including atria,ventricles, connective tissue and large vessels. Since every read had been previously assigned to a spage2vec and pciSeq-based cell type, we have calculated the frequency of every cell type/cluster on every region and computed the correlation coefficient (pearson correlation) between the cell types and clusters described in the text. Indeed, their correlation coefficient agrees with the description given in the previous version of the manuscript. Results of this analysis have been included in the manuscript in S2 Figure and referenced accordingly in the text.

1g) The authors postulate that the spage2vec results are more inline with expected distributions of cell types (line 132-133). Please provide evidence for this, especially in light of point 1b.

Despite some evidences such as the work presented by Sylven et al.(2) postulate that the spage2vec results better represent the true diversity within cardiomyocytes, currently there is no gold standard or consensus about the distribution of the different cell types, especially when referring to subpopulations. As a consequence, we have excluded the sentence postulating that the results are more in line with the expected distribution.

Intermediate

2. Is spage2vec a “graph convolutional neural network”? While there are elements of graphs, convolutional operations, and neural networks in the implementation, the authors should revisited this to see whether their description fits with the current level of precision in defining tools in the field. Interestingly, the word “convolution” does not appear in the original spage2vec publication.

Graph neural networks are the generalized version of convolutional neural networks (used on image data) for not structured data, where the numbers of nodes connections vary and the nodes are unordered, as compared to an image where the pixels follow a clear Euclidean structure. Graph neural networks are by default convolutional in the sense that they aggregate local information rather than being fully connected. Although it is equally correct to use the term ‘graph convolutional neural network, and ‘graph neural network’, we wish to keep the term ‘convolutional’ to emphasize the localized accumulation of data. We have added the word ‘convolutional’ to other parts in the text where graphs are mentioned, to be consistent with the title of the paper.

3. The methods are well described, but lack any information of computational frameworks/tools/versions that are used. For example, the authors do not describe whether their analysis is performed in matlab/R/Python/etc for numerous sections. In fairness, the authors provide access to a very good Jupyter notebook in their github repository, but (i) the link to the repository it not a DOI (see <https://docs.github.com/en/repositories/archiving-a-github-repository/referencing-and-citing-content>), and (ii) the authors and editors should decide on the level of appropriate reporting of computational tools (e.g. for NPG, I would typically list the version of tools and libraries within the methods text)

We have now added a link to a doi for the general spage2vec code and clarified that it is available as python code in a Jupyter Notebook, see 10.5281/zenodo.6591405 , see Materials and Methods.

4. It would be interesting to see how spage2vec compares to results obtained by HMRF based methods (e.g. <https://dx.doi.org/10.1038%2Fnbt.4260>, <https://doi.org/10.1038/s41587-021-01044-w>) for this dataset. While this would be interesting, a systematic comparison this may fall out of scope of the current study, so perhaps the authors could comment on how they expect their method would hold up against these other models.

Baysor creates a feature descriptor (i.e. embedding) for each spot by linearly aggregating information from its neighbors by counting the number of different molecules of each genes that are present in the node neighborhood. While spage2vec uses graph convolution layers and thus has the power to propagate and non-linearly transform information from further away in the graph and thus being able to better model sparse datasets with relatively low count of molecules per cell, as the one analyzed generated with the ISS protocol. Indeed, Baysor is not suited for very sparse spatial transcriptomic dataset like ISS (as also the authors state in the manuscript).

Instead HMRF approaches are based on computational and memory intensive optimizations, that are prohibitive for segmentation-free analysis where the spatial graph contain millions of node and billions of edges.

We believe further comparison between these methods, despite being a really interesting benchmarking for the field, is out of the scope of this work.

5. The human heart contain multi-nucleated cells, that may not be amenable to microfluidics based scRNAseq. Can the authors comment on the extent to which they expect this to be an issue in the scRNAseq data analysis, and how this may potentially link to cardiomyocyte diversity identified by spage2vec?

As the reviewer comments, muti-nucleated cells might not be amenable to microfluidics-based scRNAseq. However, the current data is from up to 9pcw, and binucleations occurs much later in the heart development close to birth (4) .Therefore, while mature cardiomyocytes are multinucleated, developmental cardiomyocytes are mononucleated and would not suffer from this limitation.

6. Why do the authors only find “immune” cells in the 6.5PCW dataset?

The immune signature, characterized by the expression of STMN2 and ISL1, among other markers, was very easy to distinguish in one of the pcw6.5 clusters, while it wasn’t that clear in the other two time points due to lack of signals. However, by re-annotating the data, we have been able to identify clusters pcw4-21 and pcw9.5-28 as immune cells. Both clusters were localized close to the immune cells from pcw-6.5 in the UMAP presented in Figure S5 and, while pcw9-28 wasn’t annotated, pcw4-21 was previously annotated as subepicardial. This is because, while some of the cells assigned to this cluster clearly express STMN2 and ISL1, other cells present a signature closer to fibroblasts, which suggests that we might be underclustering these cells. All reannotated clusters have now been updated in the Figures.

7. Datasets. There isn’t a link to the datasets that were analysed in the paper. On the github repo, the link to the raw data seems broken: <https://doi.org/10.5281/zenodo.5060858>.

The data repository has now been made public and it is now accessible through the activated doi.

Minor (these may be suggestions or questions):

8. Subheadings in the results would make is easier to read..

We have added subheadings within the results section to make it easier to read.

9. As a general minor issue, the paper reads like “I used tool X on dataset Y”, with spage2vec being mentioned a few too many times (it sometimes appears three times within a paragraph)

We have rephrased some of the sentences where spage2vec was used to improve readability.

10. The abstract could perhaps also mention their trajectory analysis results.

We have now modified the abstract to mention the trajectory analysis results.

11. Line 50. “Cell Atlasing” -> “cell atlasing”.

This typo has been corrected.

12. Line 55. Should “Single cell” be “Single cell RNAseq”?

This typo has been corrected in the current version of the manuscript.

13. Line 56. Are citations 5-9 appropriate? Given the large number of SRT methods, perhaps one or 2 review papers should be cited?

Thanks for the suggestion. We have now modified the references including some broader review papers summarizing the large number of SRT methods currently available.

14. Line 68-69. I cannot see what this states in addition to lines 67-68.

Lines 68-69 aim to serve as a one sentence summary of the pciseq algorithm, which complements the introduction to the method exposed in lines 68-69

15. Line 70. The use of “comprehensive” together with “spatial atlas” here is questionable (… to be clear Asp et al was a great study, but we should be precise with what the results were). Are the authors sure that there were no other (non-transcriptomics) imaging based analysis of the human heart? Likewise, while in the “spatial atlas” is synonymous with “spatial transcriptional atlas” in many field of science, in other fields it could mean something different (e.g. “spatial proteomic atlas”, or atlasing using radiological approaches). The authors should either tone down “comprehensive”, or rephrase the result (e.g. “spatial transcriptional atlas”/”spatial cellular atlas”).

The sentence has been rewritten to accurately describe the fact that it was the first spatial transcriptional atlas of human heart development to avoid confusion.

16. E.g. Line 85. Should “reads” be replaced for something more generic, to also be applicable for, e.g. FISH based molecule detection? The nature of this problem is not specific to ISS, but to all single molecule resolved SRT assays.

We don’t see an issue in using read to describe every decoded signal, so we’d like to keep it as it is to avoid confusion to readers familiarized with this concept.

17. Line 91. “Powerful” – is this hyperbole? Graph-based approaches are indeed useful, but the use of “powerful” does not add to the sentence.

The word powerful has been removed to avoid confusion

18. Line 149. The differences in the number of cells in the ISS and scRNAseq data should be enumerated.

We have included the numbers of cells analyzed with both methods, as requested. Note that the cells included for ISS refers to the number of cells annotated in the original Asp. et al. study, since spage2vec can’t, by definition, provide this information.

19. Line 383. “e.i.” should be “i.e.”

The typo reported has been corrected in the updated version of the manuscript.

Figures

20. Please check that figures are shown consistently. E.g Figure S4 shows the 6.5 PWC image, but this is flipped compared figure 1D.

The figures have been revised and corrected to show consistently the spatial maps.

21. Figure 1C. The scale bar has no units.

The scale bar has been modified to facilitate the interpretation of the heat map. We have also modified the caption to add information about the units used.

22. Figure 2B. The scale bar has no units. The x-axis label should be improved, e.g. “PCW6.5 spage2vec clusters”.

In the updated version of the manuscript, both the x-axis label and the scale bar units have been updated.

23. Figure 3A. Abbreviation “cl” is not explained. Perhaps add “Cluster” as the top axis label?

Figure 3A has been modified according to reviewer’s suggestions in previous points. The unexplained abbreviation “cl” is now explained in the figure legend.

24. Figure 3B. X-axis labelling is inconsistent.

Thanks for reporting it. We have now relabelled the axes to be consistent between the panels in Figure 3.

25. Figure S1, S2, S3. It would be nice if the panels were arranged by biological grouping.

Figures S1,S2 and S3 have been removed from the current version of the manuscript. The data previously represented in these figures can be interactively explored in the viewer provided with the manuscript (<https://tissuumaps.dckube.scilifelab.se/human_heart.tmap?path=demo>). As a consequence, all Supplementary figures from S4 have a new number assigned.

26. Figure S5. Perhaps this should be a main figure.

Thanks for the suggestion. We have though given priority to other panels in the main figures which we think that summarize better the results presented.

27. Figure S6. No units on the scale bar. X-axis label should be “spage2vec cluster”

We have modified the figure to add a more informative X-axis and Y-axis label. We have also included units on the scale bar.

**Additional figures**

**Additional Figure 1. A.** UMAP showing the cell types (left) identified in the scRNAseq (pcw6.5) from Asp et al. and the newly identified clusters (right) with the entire set of genes (up) or only the genes included in the ISS panel (down). **B.** Heat map representing the confusion matrix between the cell types annotated in the original work by Asp et al. and the newly identified clusters using all the genes. **C.** Heat map representing the confusion matrix between the cell types annotated in the original work by Asp et al. and the newly identified clusters using only the genes included in the ISS panel.

**References**

1.	Becht E, McInnes L, Healy J, Dutertre CA, Kwok IWH, Ng LG, et al. Dimensionality reduction for visualizing single-cell data using UMAP. Nat Biotechnol. 2019 Jan 1;37(1):38–47.

2.	Sylvén C, Wärdell E, Månsson-Broberg A, Cingolani E, Ampatzis K, Larsson L, et al. High cardiomyocyte diversity in human early prenatal heart development. Available from: https://doi.org/10.1101/2022.02.26.482029

3.	Asp M, Giacomello S, Larsson L, Wu C, Fürth D, Qian X, et al. A Spatiotemporal Organ-Wide Gene Expression and Cell Atlas of the Developing Human Heart. Cell. 2019;179(7):1647-1660.e19.

4.	Paradis AN, Gay MS, Zhang L. Binucleation of cardiomyocytes: the transition from a proliferative to a terminally differentiated state. Drug Discov Today. 2014 May 1;19(5):602–9.
